# Validation of a genome-wide association study implied that *SHTIN1* may involve in the pathogenesis of NSCL/P in Chinese population

**DOI:** 10.1038/srep38872

**Published:** 2016-12-23

**Authors:** Yirui Wang, Yimin Sun, Yongqing Huang, Yongchu Pan, Aihua Yin, Bing Shi, Xuefei Du, Lan Ma, Feifei Lan, Min Jiang, Jiayu Shi, Lei Zhang, Xue Xiao, Zhongwei Zhou, Hongbing Jiang, Lin Wang, Yinxue Yang, Jing Cheng

**Affiliations:** 1 Department of Biomedical Engineering, Medical Systems Biology Research Center, Tsinghua University School of Medicine, Beijing 100084, China; 2 CapitalBio Corporation, Beijing 102206, China; 3 National Engineering Research Center for Beijing Biochip Technology, Beijing 102206, China; 4 The State Key Laboratory Breeding Base-Shenzhen Key Laboratory of Chemical Biology, The Graduate School at Shenzhen, Tsinghua University, Shenzhen 518055, China; 5 Department of Oral and Maxillofacial Surgery, Affiliated Stomatological Hospital, Ningxia Medical University, Yinchuan 750004, China; 6 General Hospital of Ningxia Medical University, Yinchuan 750004, China; 7 National Engineering Research Center for Beijing Biochip Technology, Sub-center in Ningxia, Yinchuan 750004, China; 8 Jiangsu Key Laboratory of Oral Diseases, Nanjing Medical University, Nanjing 210029, China; 9 Medical Genetic Center, Guangdong Women and Children Hospital, Guangzhou 511442, China; 10 Maternal and Children Metabolic-Genetic Key Laboratory, Guangdong Women and Children Hospital, Guangzhou 511442, China; 11 Biobank of Guangdong Women and Children Hospital, Guangzhou 511442, China; 12 The State Key Laboratory of Oral Diseases, Sichuan University, Chengdu 610041, China; 13 West China College of Stomatology, Sichuan University, Chengdu 610041, China; 14 Division of Growth and Development and Section of Orthodontics, School of Dentistry, University of California, Los Angeles, Los Angeles, CA 90095, USA.

## Abstract

Orofacial clefts are among the most common birth defects in humans worldwide. A large-scale, genome-wide association study (GWAS) in the Chinese population recently identified several genetic risk variants for nonsyndromic cleft lip with or without cleft palate (NSCL/P). We selected 16 significant SNPs from the GWAS I stage (*P *< 1.00E-5) that had not been replicated to validate their association with NSCL/P in 1931 NSCL/P cases and 2258 controls. Ultimately, we identified a NSCL/P susceptibility loci (*rs17095681* at 10q25.3, intron of *SHTN1* and 27.2 kb downstream of *VAX*1, *P*_meta_ = 3.80E-9, OR = 0.64) in Chinese Han and Hui populations. This locus was not high LD with the reported loci in 10q25.3. It was a newly identified independent locus in 10q25.3 associated with NSCL/P. These results imply that *SHTIN1* may involve in the pathogenesis of NSCL/P advance our understanding of the genetic susceptibility to NSCL/P.

Nonsyndromic cleft lip with or without cleft palate (NSCL/P) is one of the most common birth defects with heterogeneous etiologies, including both genetic and environmental factors and their joint effects^1^. Linkage and association analyses have identified a number of candidate genes and chromosomal loci that may be associated with the risk for NSCL/P^1^,^2^. However, mutations and/or polymorphisms in these genes can only explain a small fraction of the genetic contribution to the pathogenesis of this structural abnormality due to genetic heterogeneity and gene-environment interactions. The only gene with a confirmed role in CL/P etiology across multiple populations is *IRF6*. The protein encoded by *IRF6* is a key determinant of the keratinocyte proliferation-differentiation switch and the formation of oral periderm^3^,^4^. A functional variant of *IRF6* (*rs642961*), located within the promoter sequence and disrupting the binding site for the transcription factor AP2a, also significantly increases the risk for NSCL/P^5^.

With recent advances in high-density single nucleotide polymorphism (SNP) genotyping arrays and statistical methodology, genome-wide association studies (GWAS) have heralded a new era of gene discovery for complex diseases. To date, five GWASs and one genome-wide meta-analysis on NSCL/P have been performed, which identified 13 loci or genes (8q24, *IRF6, MAFB, ABCA4, NOG, VAX1, PAX7, EPHA3, THADA, SPRY2, TPM1*, 8q21.3 and *CREBBP*)^6^,^7^,^8^,^9^,^10^,^11^ that exceeded genome-wide significant levels. It has been shown that the 8q24 region harbors remote cis-acting enhancers that control *Myc* expression in the developing face. Deletion of this regulating interval in mice results in mild alteration of facial morphology, including CL/P^12^. Most of these GWASs used samples of European origin, with the exception of Beaty *et al*. in 2010 and Sun *et al*. in 2013, which used Asian samples including Chinese samples.

The GWAS performed by Sun *et al*. in a large scale of Chinese population identified several risk genetic variants for NSCL/P^11^. In this project, Two GWASs were performed, validating 30 loci that were significant in both GWAS studies. However, some loci that exceeded the significance threshold (P < 1E-5) in the first GWA study but were not significant or imputed successfully in the second GWA study have yet to be validated. In this paper, we selected 16 such SNPs and validated them in Chinese population to further identify susceptibility loci/genes for NSCL/P. We identified one locus showing significant association with NSCL/P risk.

## Results

### Validation results

Sixteen SNPs were selected for validation based on a stepwise series of criteria (see Methods), none of which were in high LD with each other. We then performed the validation of these 16 SNPs in 1668 Chinese Han cases and 1924 Chinese Han controls from multiple hospitals in China. Three SNPs showed evidence of association with NSCL/P (*P*_meta_ < 0.05, Supplementary Table S1), but the association direction of two of these SNPs was not in concordance with the GWAS stage. One SNPs, *rs17095681* at 10q25.3 (*P*_meta_ = 8.50E-05, OR = 0.70) displayed consistent association with NSCL/P, and the significant direction was in concordance with the GWAS stage (Table 1; Supplementary Table S1). We then validated rs17095681 in Chinese Hui population, which is a minority of China, including 263 case and 334 control from General Hospital of Ningxia Medical University. The validation result was close to significance (*P* = 6.42E-2) and the association direction was in concordance with the GWAS stage (OR = 0.64). The Meta analysis *P*-values of Han and Hui population validations was 1.50E-5 (Table 1). In the combined analysis of GWAS and validation stages, *rs17095681* showed strong evidence of association (*P*_meta_ = 3.80E-9, OR = 0.64), which reached the genome-wide significance level among the Chinese Han and Hui samples (*P*_meta_ < 5.00E-8, Table 1). Another reported significant locus in 10q25.3, *rs7078160*, was also validated in these validation samples^11^. To test the independence of these two SNPs, We fix one SNP as conditional factor and analysis the association between the other SNP and NSCL/P. The conditional analysis results indicated that the effect of *rs17095681* was not correlated with *rs7078160* (*P* value of *rs17095681* was *P*_meta_ = 7.82E-4 conditioned on *rs7078160*, Supplementary Table S2). The LD analysis indicated that rs17095681 was not linkage with rs7078160 in four validation cohort (r^2^ < 0.1, Supplementary Table S3). These results indicated that rs17095681 may be an independent locus associated with NSCL/P at 10q25.3 in Chinese Han and Hui populations.

### Analysis of different genetic models

For the significant SNP *rs17095681*, we used other genetic models, the additive model, allelic model and genotypic model for further analysis. We observed that this locus significant under the dominant model achieved similar results under the additive model (*P*_meta_ = 8.96E-5), allelic model (*P*_meta_ = 2.56E-5) and genotypic model (*P*_meta_ = 9.85E-6) in validation stage (Table 2). In the combined analysis of the two stages, this locus achieved similar results with dominant model under the genotypic (het) model (*P*_meta_ = 3.40E-9). It also achieved near genome-wide significance in additive (*P*_meta_ = 6.50E-8) and allelic model (*P*_meta_ = 5.65E-8). In Summary, the SNP rs17095681 was associated with NSCL/P significantly in additive model, allelic model and genotypic model.

## Discussion

**Figure 1:**
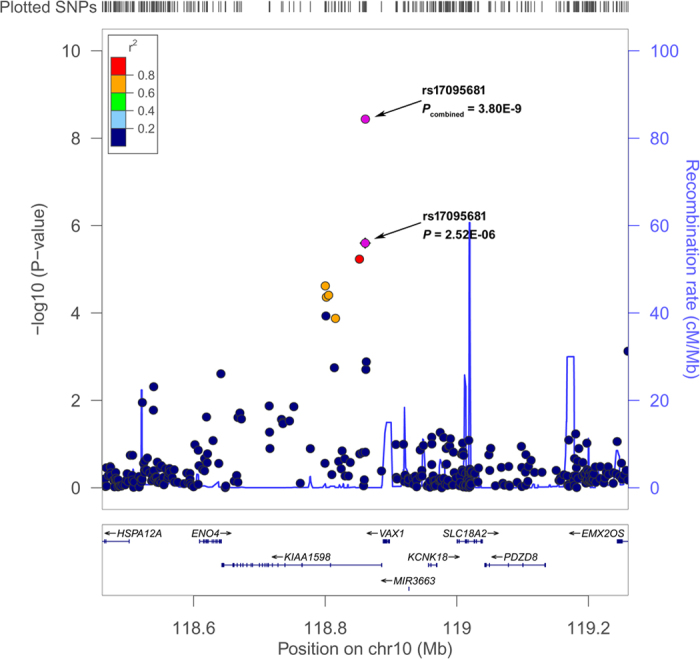
Regional plots of the susceptibility locus rs17095681. Regional plots of newly discovered locus rs17095681 associated with the risk for NSCL/P in a Chinese population in the GWAS discovery stage. The results (−log_10_ *P, P*-value of GWAS) are shown for SNPs in the region 400 kb upstream or downstream of the marker SNP. The marker SNP is shown as a purple diamond in the GWAS stage and as a purple circle in the combined stage. The LD values (r^2^) between the SNPs and the most strongly associated SNP (diamond), rs17095681 are indicated by the heat scale. The genes within the region of interest are annotated, and the directions of transcripts are shown in arrows.

In this validation study of NSCL/P, we identified one SNP at 10q25.3, *rs17095681*, which were significantly associated with NSCL/P risk in Chinese Han and Hui populations. It reached the genome-wide significance threshold (*P*_meta_ = 3.40E-9) in the combined analysis. The SNP *rs17095681* is located in the intron of *SHTN1* (also known as KIAA15598) and 27.2 kb downstream of *VAX1* (ventral anterior homeobox 1, Fig. 1). It has been reported that four SNPs with P_GWAS_ < E-4 are located in a 30-kb region that is 50 kb downstream of VAX1^8^. The SNP rs17095681 is not in high LD (r^2^ > 0.8) with these SNPs. Rs7078160 has been successfully validated in a Chinese population^11^. The SNPs *rs17095681* and *rs7078160* are 33 kb apart from each other, but the effect of *rs17095681* is not correlated with *rs7078160* (Supplementary Table S2), indicating that *rs17095681* is in an independent block associated with NSCL/P.

*SHTN1* code a linker molecule shootin 1 that couples F-actin retrograde flow and the cell adhesion molecule (CAM) L1-CAM^13^ at neuronal growth cones to promote neuronal polarization and axon outgrowth. The attractive axon guidance molecule netrin-1^14^,^15^ induces Pak1-mediated shootin1 phosphorylation in axonal growth cones^16^ which in turn enhances the coupling between F-actins and shootin1, thereby promoting the traction forces for axon outgrowth. It has been reported that netrin-1 gene *NTN1* is associated with NSCL/P^9^,^11^,^17^. *NTN1* encodes the protein NETRIN 1, which plays a role in the developing the nervous system by promoting both axonal outgrowth and axonal guidance in pathfinding^18^,^19^,^20^,^21^. In addition, NTN1 was up regulated in dental pulp stem cell cultures from NSCL/P patients^11^. This information suggested that *SHTIN1* and *NTN1* play important roles in the development of NSCL/P.

The other gene near SNP *rs17095681, VAX1* was also been reported associated with NSCL/P. Mice with homozygous Vax1 mutations display craniofacial malformations including cleft palate^22^. Two individuals with a 10q terminal deletion syndrome with breakpoints in 10q25 have been reported, one with a submucous cleft palate^23^ and the other with a cleft lip^24^.

In summary, in this validation of a NSCL/P GWAS in Chinese populations, we identified a susceptibility locus at 10q25.3 that reached genome-wide significance. The *rs1709568* SNP at 10q25.3 is located in an independent block and therefore is not related to any previously reported associated loci. Genes near this locus participate in the processes of neuronal axon outgrowth and cell migration. It is known that cell migration are crucial for oralfacial development. Further studies with larger sample sizes are warranted to replicate our findings. Fine mapping around this locus and related functional studies should also be performed to elucidate the molecular mechanisms underlying the observed associations.

## Methods

### Study populations

We performed the validation study using samples from four regions in China, including 1931 NSCL/P cases and 2258 controls. A summary of all cases and controls in the study is provided in Table 3. All of the samples were unrelated individuals of Chinese descent, obtained from Guangdong Maternal and Child Health Care Hospital (validation a, 497 Chinese Han cases and 497 Chinese Han controls for validation), Western China Hospital of Stomatology Sichuan University (validation b, 480 Chinese Han cases and 482 Chinese Han controls, independent with GWAS samples), the Institute of Stomatology, Nanjing Medical University (validation c, 446 Chinese Han cases, 522 Chinese Han controls, independent with GWAS samples), and General Hospital of Ningxia Medical University (validation d, 245 Chinese Han cases, 423 Chinese Han controls; validation e, 263 Chinese Hui cases and 334 Chinese Hui control). All cases were recruited in local hospitals and independently confirmed as NSCL/P by two gynecologic pathologists during routine diagnosis. Syndromic cleft lip or palate patients and cleft palate-only patients were excluded. Controls were recruited in local hospitals for individuals receiving routine physical examinations or healthy newborns whose parents volunteered to donate their umbilical cord blood. All controls were clinically assessed to be without cleft lip or palate or family history of cleft lip or cleft palate (including first, second, and third degree relatives). The cases and controls were frequency-matched for age and gender. At recruitment, informed consent was obtained from each subject. This study was approved by the ethics committees of Guangdong Maternal and Child Health Care Hospital, Western China Hospital of Stomatology Sichuan University, the Institute of Stomatology, Nanjing Medical University and General Hospital of Ningxia Medical University and the methods were carried out in accordance with the approved guidelines.

### SNP selection and genotyping

SNPs for the replication stage were selected using the following criteria: (i) SNPs with *P *< 1.00E-5 in the first GWA study but were not significant or imputed successfully in the second GWA study; (ii) only the SNP with the lowest P-value was selected when multiple SNPs were observed but in strong linkage disequilibrium (LD) (r^2^ > = 0.8); (iii) primers could be successfully designed using Sequenom primer design software; and (iv) SNPs had not previously been validated. A total of 16 SNPs that matched these criteria were included in the replication stage. Genotyping of replicates was conducted by the Sequenom MassARRAY system at Beijing CapitalBio Technology Company, Beijing, China.

### Quality control at the replication stage

We excluded SNPs with a call rate < 90% or a deviation from Hardy-Weinberg equilibrium (*P *< 0.05) in the controls. All 16 SNPs exceed quality control and were used for further analysis.

### Association analysis in the replication and combined stage

For the replication studies, associations between SNP genotypes and disease status were assessed in a dominant model in PLINK v1.07 (http://pngu.mgh.harvard.edu/Bpurcell/plink/) using logistic regression modeling with gender as a covariate. Joint analyses of all combined samples at the validation stage and GWAS stage were conducted by using either the random effects model (I^2^ > 25%) or by using the fixed-effect model (I^2^ < 25%). Another genetic model (the additive, allelic and genotypic model) was also calculated for the associated SNPs. The chromosome regions of significant loci were plotted using an online tool, LocusZoom 1.1 (http://csg.sph.umich.edu/ locuszoom/).

## Additional Information

**How to cite this article**: Wang, Y. *et al*. Validation of a genome-wide association study implied that *SHTIN1* may involve in the pathogenesis of NSCL/P in Chinese population. *Sci. Rep.* **6**, 38872; doi: 10.1038/srep38872 (2016).

**Publisher's note:** Springer Nature remains neutral with regard to jurisdictional claims in published maps and institutional affiliations.

## Supplementary Material

Supplementary File

## Figures and Tables

**Table 1:** Significant associations of the SNP with NSCL/P risk in the entire sample.

Chr.	SNP	Stage	Region	MAF		Case	Control	OR (CI 95%)	*P*	*P*_meta_^*^	*P*_meta**_	*P*_meta_^#^
				Case	Control							
10	rs17095681	GWAS	Sichuan	0.064	0.123	59/439	100/330	0.42 (0.29–0.60)	2.52E-6			3.80E-9
		Validation a	Guangdong	0.070	0.113	64/431	107/389	0.53 (0.38–0.75)	2.91E-4	8.50E-5	1.50E-5	
		Validation b	Sichuan	0.081	0.097	71/405	84/395	0.82 (0.58–1.16)	2.67E-1			
		Validation c	Nanjing	0.097	0.104	79/368	105/412	0.84 (0.61–1.17)	2.99E-1			
		Validation d	Ningxia (Han)	0.077	0.115	36/211	89/328	0.62 (0.40–0.95)	2.71E-2			
		Validation e	Ningxia (Hui)	0.059	0.090	31/231	58/275	0.64 (0.40–1.03)	6.42E-2			

Associations are shown for the discovery, replication, and combined samples.

^*^Meta analysis *P*-value of Han population validations.

^**^Meta analysis *P*-value of Han and Hui population validations.

^#^Meta analysis *P*-value of the GWAS and validation stage.

**Table 2:** Genetic model analysis for the significant SNP in the validation samples.

Chr.	SNP	Stage	OR (95% CI)			*P*			*P*_meta_*			*P*_meta_#		
			Additive	Allelic	Genotypic (het)	Additive	Allelic	Genotypic (het)	Additive	Allelic	Genotypic (het)	Additive	Allelic	Genotypic (het)
10	rs17095681	GWAS	0.47 (0.34–0.66)	0.49 (0.17–0.35)	0.43 (0.19–0.30)	9.09E-6	1.19E-5	6.34E-6				6.50E-8	5.65E-8	3.40E-9
		Validation a	0.59 (0.43–0.81)	0.59 (0.16–0.43)	0.51 (0.18–0.36)	1.09E-3	1.01E-3	1.94E-4	8.96E-5	2.56E-5	9.85E-6			
		Validation b	0.83 (0.61–1.12)	0.82 (0.16–0.60)	0.84 (0.18–0.58)	2.28E-1	2.28E-1	3.34E-1						
		Validation c	0.84 (0.61–1.17)	0.93 (0.15–0.69)	0.77 (0.17–0.55)	6.54E-1	6.50E-1	1.29E-1						
		Validation d	0.65 (0.44–0.96)	0.65 (0.20–0.44)	0.64 (0.22–0.41)	2.96E-2	3.02E-2	4.47E-2						
		Validation e	0.62 (0.39–0.99)	0.64 (0.23–0.41)	0.67 (0.24–0.41)	4.40E-2	4.84E-2	9.59E-2						

Associations are shown for the discovery, replication, and combined samples.

^*^Meta analysis *P*-value of validations.

^#^Meta analysis *P*-value of the GWAS and validation stage.

**Table 3:** Sample characteristics of cases with NSCL/P and controls.

Variables	GWAS		Validation									
	(Sichuan)		Valiidation a (Guangdong)		Validation b (Sichuan)		Validation c (Nanjing)		Validation d (Ningxia Han)		Validation e (Ningxia Hui)	
	Case (n = 504)	Control (n = 455)	Case (n = 497)	Control (n = 497)	Case (n = 480)	Control (n = 482)	Case (n = 446)	Control (n = 522)	Case (n = 245)	Control (n = 423)	Case (n = 263)	Control (n = 334)
**Gender**												
Male	308	236	330	287	310	280	273	333	162	252	170	164
Female	196	219	167	210	170	202	173	189	83	171	93	170
