# Supplementary material for: Validation of a genome-wide association study implied that SHTIN1 may involve in the pathogenesis of NSCL/P in Chinese population
Source: Sci Rep. 2016 Dec 23;6:38872. doi: 10.1038/srep38872 (PMC5180092; doi:10.1038/srep38872)
Supplement: Supplementary File [file srep38872-s1.doc]

**Validation of a genome-wide association study implied that *SHTIN1* may involve in the pathogenesis of NSCL/P in Chinese population**

Yirui Wang^1,2,3,#^, Yimin Sun^1,2,3,4,#^, Yongqing Huang^5,6,7,#^, Yongchu Pan^8,#^, Aihua Yin^9,10,11,#^ Bing Shi^12,13^, Xuefei Du^5,6^, Lan Ma^8^, Feifei Lan^9,10,11^, Min Jiang^6,7^ Jiayu Shi^14^, Lei Zhang^2,3^, Xue Xiao^2,3^, Zhongwei Zhou^5,6^, Hongbing Jiang^8^, Lin Wang^8,*^, Yinxue Yang^5,6,*^ & Jing Cheng^1,2,3,*^.

**Supplementary Table S1. Associations of the 16 SNPs with NSCL/P in the GWAS scan and validation.**

| **Chr.** | **BP** | **SNP** | **Stage** | **A1** | ***OR*^a^** | ***P*^b^** | ***P*_meta_^c^** |
| --- | --- | --- | --- | --- | --- | --- | --- |
| 1 | 16420516 | rs77273558 | GWAS | C | 0.13 (0.08-0.21) | 1.17E-16 | 1.18E-04 |
|  |  |  | Validation |  | 1.16 | 3.59E-01 |  |
| 1 | 240554884 | rs80009068 | GWAS | G | 0.17 (0.11-0.26) | 3.80E-16 | 5.73E-01 |
|  |  |  | Validation |  | 1.38 | 1.46E-03 |  |
| 2 | 175200903 | rs35548534 | GWAS | C | 3.32 (2.06-5.36) | 8.99E-07 | 5.86E-03 |
|  |  |  | Validation |  | 1.09 | 4.45E-01 |  |
| 3 | 59380011 | rs7640340 | GWAS | G | 1.88 (1.42-2.48) | 8.34E-06 | 4.92E-02 |
|  |  |  | Validation |  | 1.00 | 9.87E-01 |  |
| 4 | 54810295 | rs34814914 | GWAS | C | 0.21 (0.14-0.32) | 2.21E-13 | 1.17E-02 |
|  |  |  | Validation |  | 1.12 | 3.21E-01 |  |
| 6 | 82461520 | rs3186631 | GWAS | G | 2.38 (1.75-3.24) | 3.01E-08 | 2.94E-01 |
|  |  |  | Validation |  | 0.83 | 4.49E-02 |  |
| 6 | 90543362 | rs12193964 | GWAS | C | 0.15 (0.09-0.23) | 1.39E-16 | 6.33E-07 |
|  |  |  | Validation |  | 0.89 | 4.09E-01 |  |
| 10 | 11618089 | rs117819323 | GWAS | G | 0.17 (0.11-0.25) | 9.35E-17 | 3.13E-04 |
|  |  |  | Validation |  | 1.18 | 2.47E-01 |  |
| 10 | 118860777 | rs17095681 | GWAS | T | 0.42 (0.29-0.6) | 2.52E-06 | 2.21E-08 |
|  |  |  | Validation |  | 0.70 | 8.50E-05 |  |
| 12 | 26111000 | rs74589780 | GWAS | C | 0.19 (0.13-0.28) | 1.87E-16 | 3.72E-05 |
|  |  |  | Validation |  | 0.97 | 7.88E-01 |  |
| 14 | 36262089 | rs75360196 | GWAS | G | 0.09 (0.05-0.17) | 3.51E-15 | 7.01E-04 |
|  |  |  | Validation |  | 1.23 | 2.92E-01 |  |
| 14 | 62496499 | rs115571837 | GWAS | G | 0.14 (0.09-0.22) | 6.04E-18 | 1.17E-04 |
|  |  |  | Validation |  | 1.01 | 9.35E-01 |  |
| 15 | 99526388 | rs116709483 | GWAS | G | 0.11 (0.06-0.18) | 1.18E-15 | 1.62E-05 |
|  |  |  | Validation |  | 1.05 | 8.05E-01 |  |
| 21 | 33580009 | rs76436925 | GWAS | G | 0.3 (0.19-0.49) | 1.14E-06 | 6.40E-02 |
|  |  |  | Validation |  | 1.20 | 2.72E-01 |  |
| 22 | 34105970 | rs7289968 | GWAS | C | 0.47 (0.35-0.62) | 1.96E-07 | 1.49E-03 |
|  |  |  | Validation |  | 0.93 | 3.48E-01 |  |
| 22 | 46324171 | rs77060195 | GWAS | G | 0.15 (0.07-0.29) | 9.13E-08 | 9.18E-05 |
|  |  |  | Validation |  | 0.74 | 8.84E-02 |  |

a OR of GWAS stage or meta-analysis of the validation stage.

b *P-*value of the GWAS stage or meta-analysis of the validation stage.

c *P-*value of the meta-analysis of two stages.

**Supplementary Table S2. Conditional analysis of the two 10q25.3** loci.

| SNP | Controled SNP | Validation a | | |  | Validation b | |  | Validation c | |  | | Validation d | | | |  | Validation e | | |  | Validation combined | | |  |
| --- | --- | --- | --- | --- | --- | --- | --- | --- | --- | --- | --- | --- | --- | --- | --- | --- | --- | --- | --- | --- | --- | --- | --- | --- | --- |
|  |  | OR^a^ | *P* ^a^ | |  | OR^a^ | *P* ^a^ |  | OR^a^ | *P*^a^ |  | | OR^a^ | | *P* ^a^ | |  | OR^a^ | *P* ^a^ | |  | OR^a^ | | *P* ^a^ |  |
| *rs17095681* |  | 0.53 | 2.91E-04 | |  | 0.82 | 2.67E-01 |  | 0.84 | 2.99E-01 |  | | 0.63 | | 2.71E-02 | |  | 0.64 | 6.42E-02 | |  | 0.69 | | 1.50E-05 |  |
| *rs17095681* | *rs7078160* | 0.55 | | 2.05E-03 |  | 0.88 | 4.67E-01 |  | 0.84 | 3.32E-01 | |  | | 0.76 | | 2.51E-01 |  | 0.65 | | 8.97E-02 |  | 0.74 | 7.82E-04 | | |

a Conditional logistic regression analysis using validation samples for each region.

b Conditional logistic regression analysis using validation samples for all regions.

**Supplementary Table S3. Linkage disequ analysis results of rs17095681 and rs7078160**

| **Chr.** | **SNP1** | **SNP2** | **S**tag | **Region** | **r2** | **D'** |
| --- | --- | --- | --- | --- | --- | --- |
| 10 | rs17095681 | rs7078160 | Validation a | Guangdong | 0.093 | 0.964 |
|  |  |  | Validation b | Sichuan | 0.089 | 1.000 |
|  |  |  | Validation c | Nanjing | 0.091 | 0.976 |
|  |  |  | Validation d | Ningxia（Han） | 0.098 | 1.000 |
|  |  |  | Validation e | Ningxia（Hui） | 0.067 | 0.958 |
